# Oxygen supplementation facilitating successful prosthetic fitting and rehabilitation of a patient with severe chronic obstructive pulmonary disease following trans-tibial amputation: a case report

**DOI:** 10.1186/1752-1947-4-410

**Published:** 2010-12-22

**Authors:** Jasdeep Sohal, Amarjit Arneja, Sat Sharma

**Affiliations:** 1 Department of Internal Medicine, St. Boniface General Hospital, BG034, 409 Tache Avenue, Winnipeg, MB, Canada R2 H 2A6

## Abstract

**Introduction:**

Dysvascular amputations are increasingly performed in patients with underlying cardiac and pulmonary disorders. A limb prosthesis is rarely offered to patients with severe chronic obstructive pulmonary disease because of their inability to achieve the high energy expenditure required for prosthetic ambulation. We describe a case of successful prosthetic fitting and rehabilitation of a patient with severe chronic obstructive pulmonary disease with the aid of oxygen supplementation.

**Case presentation:**

A 67-year-old aboriginal woman with severe chronic obstructive pulmonary disease and hypercapnic respiratory failure underwent right trans-tibial (below the knee) amputation for severe foot gangrene. An aggressive rehabilitation program of conditioning exercises and gait training utilizing oxygen therapy was initiated. She was custom-fitted with a right trans-tibial prosthesis. A rehabilitation program improved her strength, endurance and stump contracture, and she was able to walk for short distances with the prosthesis. The motion analysis studies showed a cadence of 73.5 steps per minute, a velocity of 0.29 meters per second and no difference in right and left step time and step length.

**Conclusion:**

This case report illustrates that patients with significant severe chronic obstructive pulmonary disease can be successfully fitted with limb prostheses and undergo rehabilitation using supplemental oxygen along with optimization of their underlying comorbidities. Despite the paucity of published information in this area, prosthesis fitting and rehabilitation should be considered in patients who have undergone amputation and have severe chronic obstructive disease.

## Introduction

Every year approximately 130,000 lower-limb amputations are performed in the United States, and approximately 500 amputations are carried out in the province of Manitoba (total population of 1.1 million people) [1,2]. Rehabilitation of a patient who has undergone amputation is an intricate process, as several factors determine successful ambulation with a limb prosthesis. These factors include pre-existing pulmonary disease, cardiovascular disease, peripheral vascular disease, diabetes, hypertension, hyperlipidemia, the status of the other limb and functional level prior to amputation [3]. The level of the amputation is also a key determinant for successful ambulation. Peripheral vascular disease accounts for over 90% of all amputations, and more than half occur in people diagnosed with diabetes [4,5]. Increases in the amputation rate can be expected as both the number of patients with diabetes and the number of elderly in the general population is rising, with estimated five-year survival of 30-40% after amputation [1]. The age range of the patients who undergo dysvascular amputations in North America is between 55 and 85 years. It has been estimated that the five-year survival after amputation is 30-40% [1]. The two-year survival after lower-extremity amputations is encouraging and averages at 50-60%, with most deaths attributed to the cardiovascular complications [6]. However, there is a 20-50% risk of losing the contralateral leg to the peripheral vascular disease during the four years after amputation [7]. The more proximal levels of amputation are associated with decreased survival rates [8].

Compared to normal biped ambulation, the energy costs for prosthetic ambulation are much higher. Pinzur *et al*. [9] reported increased energy expenditure of walking with limb prostheses over normal ambulation as follows: unilateral trans-tibial amputation 40-60%, unilateral trans-femoral amputation 90 to 120%, bilateral trans-tibial amputation 60-100% and bilateral trans-femoral amputation > 200%. Patients with severe chronic obstructive pulmonary disease (COPD) are rarely offered limb prostheses as many patients are limited by their ventilatory status and unlikely to achieve the high energy expenditure required for successful prosthetic ambulation.

## Case Presentation

We report a case of a 67-year-old aboriginal woman who was admitted to the rehabilitation ward. In March 2007, the patient underwent right femoral popliteal artery bypass surgery for occlusive peripheral vascular disease. Four months later, intermittent claudication recurred; she also complained of right leg pain at rest and developed ulceration of the right toes. The patient underwent right trans-tibial amputation in August 2007 because of ischemia and gangrene of the foot. This was followed by left superficial femoral artery stent placement in November 2007. Her ankle brachial pressure index was markedly reduced at 0.11 (normal, 0.95-1.2). Despite previous surgical treatment, her peripheral vascular disease progressed to gangrene of the right foot, thus necessitating the right trans-tibial amputation. The stump healing was initially delayed because of the wound infection, but eventually healed well. Her past medical history included a 61 pack-year smoking history, severe COPD, type 2 diabetes mellitus, hypertension, ischemic heart disease and a myocardial infarction three years ago treated with percutaneous coronary intervention and stent placement. The patient had a supportive husband, lived in a wheelchair-accessible bi-level home and was using a wheelchair for ambulating long distances and was mobilized with a walker for short distances.

Her physical examination revealed a well-oriented individual with normal vital signs and oxygen saturation at 88% on room air. Her neurological and cardiac examinations were normal. The respiratory examination showed hyperinflation of the thorax, decreased air entry to the lung bases bilaterally and occasional expiratory wheezing. Her residual limb length was 5 cm from the tibial tuberosity and had a 15-degree flexion contracture. The incision line was well healed with an adherent scar. Her left lower extremity showed some atrophic changes: loss of hair with absent dorsalis pedis and posterior tibial pulses were noted. The popliteal pulse was palpable but weak. General strength was graded 4 to 4+ out of a maximum of 5 in both upper and left lower extremities. Laboratory investigations revealed pulmonary function tests showing severe irreversible airflow obstruction with the following findings on pulmonary function tests consistent with severe COPD: forced expiratory volume in 1 second (FEV1) was 0.54 L/s (25% predicted) and forced vital capacity (FVC) of 1.37 L (52% predicted). Arterial blood gases demonstrated compensated hypercapnic respiratory failure (PaCO_2 _at 54 mmHg) and hypoxemia (PaO_2 _at 58 mmHg). An echocardiogram showed a normal systolic ejection fraction at 76% with mild diastolic dysfunction.

In December 2008, the patient underwent 10 weeks of in-patient rehabilitation. Her severe COPD was optimized with inhaler therapy consisting of bronchodilators and inhaled corticosteroids. Oxygen therapy was utilized during rehabilitation exercises and ambulation, with the goal being to keep percutaneous oxygen saturation above 92% during activities and rehabilitation. Following initial slow progress due to the patient's generalized deconditioning, low endurance and stump contracture, her motivation and endurance gradually improved. She was then able to fully participate in the rehabilitation program. She attended two physiotherapy sessions per day (approximately 60 minutes in length each time). Her pre-prosthetic rehabilitation program included general upper- and lower-extremity strengthening and conditioning exercises. Oxygen supplementation during exercise and ambulation greatly facilitated the rehabilitation. We were able to improve the stump contracture from 15 degrees to 10 degrees, and she was able to hop with the aid of a walker. She was casted for a custom trans-tibial patellar tendon-bearing prosthesis with a 1.5-mm silicone liner (ICEROSS) and sleeve suspension system with a dynamic solid ankle cushion heel (SACH) foot. With further gait training, she was able to ambulate 200 feet with the aid of a walker and was discharged to home.

At the time of hospital discharge, kinematic data were collected using the VICON motion analysis system to capture the kinematics of the lower limbs and the spatio-temporal parameters of her gait. The patient walked independently with supplemental oxygen using a two-wheeled walker. It was unknown how much weight bearing occurred through the upper extremities during the level walking trials. Her cadence and velocity were very slow compared to 76- to 87-year-old community-dwelling older adults (Table 1), but there was no difference between the left and right step time and step length [10]. However, the time spent in the right single support of the gait cycle was considerably less than the time spent on the left single support of the gait cycle. At a follow-up visit at six months, the patient had returned to her previous activities. She lived independently, ambulated and performed activities of daily living with the use of her prosthesis.

**Table 1:** Kinematics of the lower limb and the spatiotemporal parameters of gaita

	Patient	76- to 87-year-old adults
Cadence (steps/min)	73.5	108
Velocity (m/s)	0.29	1.16
(L) step time (s)	0.82	
(R) step time (s)	0.82	
(L) step length (cm)	23.0	63.5
(R) step length (cm)	25.0	64.3
(L) step support (s)	0.49	
(R) step support (s)	0.37	

^a^Comparisons are shown in the last column for 76- to 87-year-old community-dwelling older adults (Menz *et al*. [8]).

## Discussion

This case highlights a satisfying functional outcome for a patient with trans-tibial amputation with severe COPD among other comorbidities, who is currently living in her own house and is participating in housework. Her successful outcome was secondary to oxygen therapy and optimization of underlying severe COPD. Patients with severe COPD are unlikely to achieve prerequisite high oxygen consumption levels for prosthetic ambulation because of ventilatory and gas exchange limitations. However, with supplemental oxygen, our patient was rehabilitated successfully.

Only a few cases of successful rehabilitation of a patient with severe COPD have been reported in the literature [11,12]. Our case highlights the point that age and other co-morbidities should not be considered a barrier to rehabilitation and prosthetic fitting in patients with limb amputations. The energy required for ambulation in trans-tibial amputation is about 40-60% above normal [9]. This energy demand becomes even higher when patients have COPD and additional significant co-morbidities. Thus rehabilitation in this population is challenging, and these patients require optimization of the underlying medical condition and close medical monitoring to avoid cardiovascular complications. Generally, patients with trans-tibial amputation, whether unilateral or bilateral, cope better than those who undergo above-knee amputation. This is particularly important in patients with COPD because preserving the knee joint helps decrease the energy demands on an already taxed cardiovascular and pulmonary system. With optimization of airflow obstruction and supplemental oxygen, it is possible to achieve the high energy consumption required of prosthetic gait ambulation and successful rehabilitation. Sioson *et al*. [11] previously reported three cases in which they demonstrated successful rehabilitation of older adults with COPD. McAnelly *et al*. [12] described a case of hip disarticulation and successful rehabilitation of an individual with COPD. None of these papers reported the use of supplemental oxygen specifically for the purpose of prosthetic ambulation. Oxygen consumption during exercise can be measured by formal cardiopulmonary exercise testing. Since these patients are incapable of performing objective testing on a bicycle ergometer or treadmill, the use of an arm ergometer is suggested. We recommend formal testing utilizing an arm ergometer and oxygen supplementation to assess whether a patient can meet the required oxygen consumption criteria during such testing. Our case highlights the importance of prospective investigations to document the benefits of supplemental oxygen during rehabilitation of patients who have undergone limb amputation and have severe underlying COPD.

## Conclusions

Patients with lower-limb amputations with severe or advanced COPD are generally not considered candidates for prosthetic fitting and rehabilitation. However, we describe a case of a 67-year-old woman with severe COPD who was fitted with a lower-limb prosthesis and successfully rehabilitated. In our opinion, patients with severe COPD should be carefully assessed, regardless of their age and preexisting respiratory disorders, and a trial period of rehabilitation should be considered to explore the possibility of prosthetic fitting. We also suggest that the use of supplemental oxygen during rehabilitation and prosthetic gait ambulation may be of additional benefit to these individuals.

## Competing interests

The authors declare that they have no competing interests.

## Consent

Written informed consent was obtained from the patient for publication of this case report. A copy of the written consent is available for review by the Editor-in-Chief of this journal.

## Authors' contributions

JS analyzed and interpreted the patient data regarding the hospital progress and rehabilitation. AA supervised the prosthetic fitting and rehabilitation. AA and SS were major contributors in writing the manuscript. All authors read and approved the final manuscript.
